# Thiamine deficiency contributes to synapse and neural circuit defects

**DOI:** 10.1186/s40659-018-0184-5

**Published:** 2018-09-19

**Authors:** Qiujian Yu, Huimin Liu, Shaoming Sang, Lulan Chen, Yingya Zhao, Yun Wang, Chunjiu Zhong

**Affiliations:** 1 Department of Neurology, Zhongshan Hospital, Fudan University, Shanghai, 200032 China; 2 Institutes of Brain Science & Collaborative Innovation Center for Brain Science, State Key Laboratory of Medical Neurobiology, Fudan University, Shanghai, 200032 China

**Keywords:** Thiamine deficiency, Synaptic dysfunction, Amyloid-β, Alzheimer’s disease

## Abstract

**Background:**

The previous studies have demonstrated the reduction of thiamine diphosphate is specific to Alzheimer’s disease (AD) and causal factor of brain glucose hypometabolism, which is considered as a neurodegenerative index of AD and closely correlates with the degree of cognitive impairment. The reduction of thiamine diphosphate may contribute to the dysfunction of synapses and neural circuits, finally leading to cognitive decline.

**Results:**

To demonstrate this hypothesis, we established abnormalities in the glucose metabolism utilizing thiamine deficiency in vitro and in vivo, and we found dramatically reduced dendrite spine density. We further detected lowered excitatory neurotransmission and impaired hippocampal long-term potentiation, which are induced by TPK RNAi in vitro. Importantly, via treatment with benfotiamine, Aβ induced spines density decrease was considerably ameliorated.

**Conclusions:**

These results revealed that thiamine deficiency contributed to synaptic dysfunction which strongly related to AD pathogenesis. Our results provide new insights into pathogenesis of synaptic and neuronal dysfunction in AD.

## Background

Alzheimer’s disease (AD) is the most prevalent type of dementia, causes progressive cognitive impairment and devastating burden for individuals and their families [1]. Excepting major pathological hallmarks: senile plaques and neurofibrillary tangle, synaptic dysfunction is an early pathological event in AD correlates strongly with cognitive deficit and disease progression [2]. The amyloid cascade hypothesis postulates that amyloid-β (Aβ) peptide deposition initiates neurofibrillary tangles formation, synaptic dysfunction, and neurodegeneration, which ultimately causes cognitive deficit. Despite extensive evidence supports Aβ-driven synaptic dysfunction, clinical treatment to reduce Aβ deposition failed to reverse or to halt cognitive decline, even a considerable reduction in Aβ deposition. An increasing number of studies suggests that cognitive decline is the result of interplays among multiple pathophysiologies and that targeting Aβ alone may not be sufficient to treat Alzheimer’s disease. Instead, to clarify the other mechanisms underlying synaptic dysfunction may yield insights into new therapeutic strategies towards ameliorating cognitive decline.

Thiamine diphosphate (TDP), the active form of thiamine, is a critical co-enzyme of three key enzymes in glucose metabolism: Pyruvate dehydrogenase and α-ketoglutarate dehydrogenase in the Kreb cycle, and transketolase in the pentose phosphate pathway. The evidence suggest that there is a close association between AD and thiamine deficiency. Glod and his colleagues detected the plasma thiamine levels in AD and PD patients, and found a dramatically decreased thiamine concentration in AD subjects but not in PD invidious [3]. Several target genes have revealed the connection between AD and thiamine deficiency, such as apolipoprotein E (ApoE), p53, glycogensynthetase kinase-3β (GSK-3β), transketolase, etc. [4]. In addition, the activities of thiamine-related enzymes, the levels of thiamine and thiamine-associated isoforms are reduced in the brain tissues of AD patients [4]. Our and other’s previous studies have well demonstrated that the reduction of TDP level strongly correlates with brain glucose metabolism in AD patients [3, 5–9].

As one of the main pathological characteristics in AD, synaptic dysfunction has been well demonstrated in culturing neurons and TD mice. The loss of synapses appears to be closely correlated with the degree of cognitive deficit of AD patients [10].

Together, TDP reduction may contribute to the dysfunction of synapses and neural circuits and finally lead to the cognitive decline.

## Results

### Thiamine deficiency induced spine density decrease in vitro and in vivo

**Fig. 1:**
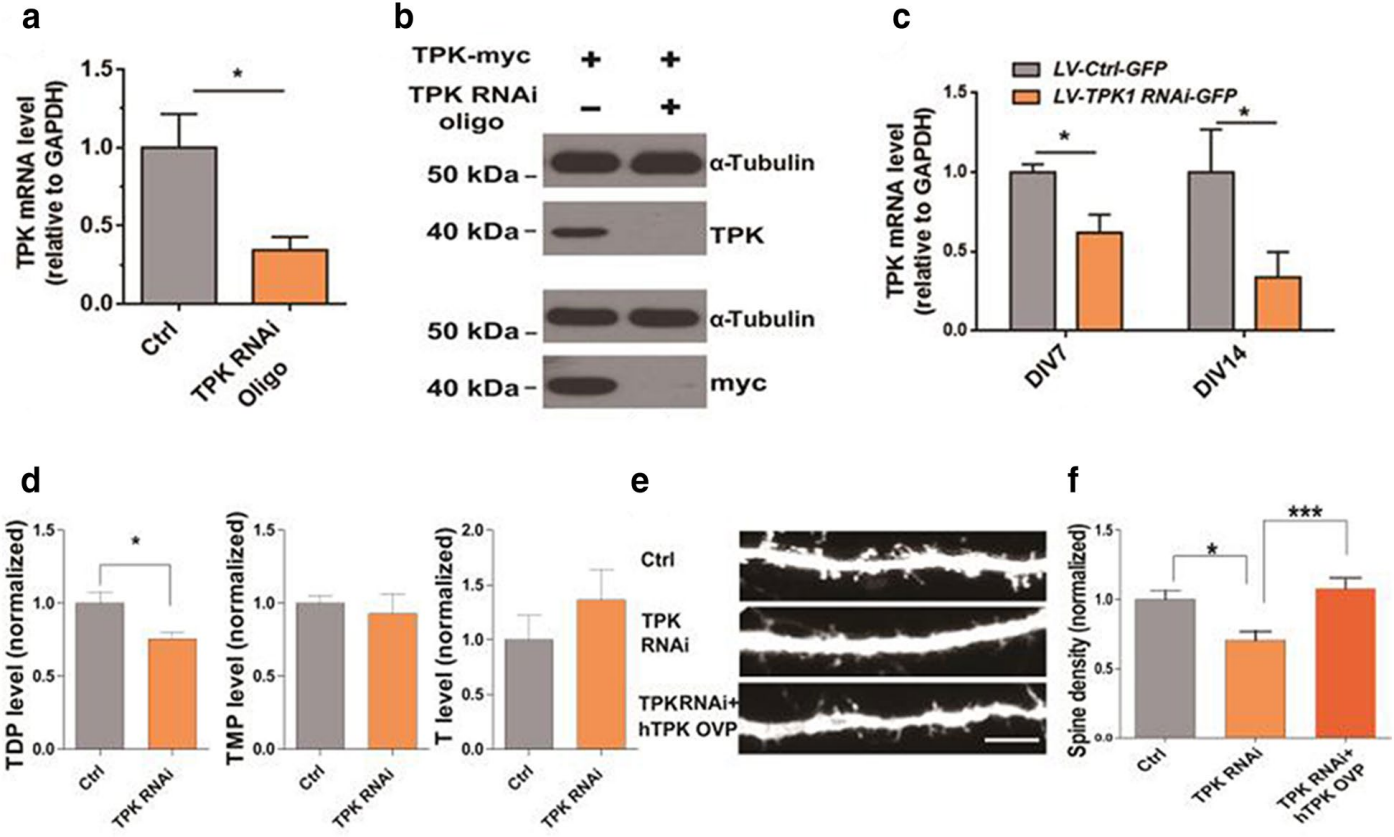
Thiamine deficiency induced spine density decrease in vitro. **a** TPK mRNA level was significantly reduced in TPK RNAi oligo transfected N2A cell lines (N = 3 culture preparations). **b** TKP expression examined by western blot in N2A cell lines. TPK overexpression was significantly reduced by RNAi, demonstrating efficiency of the RNAi at the protein level. **c** TPK mRNA level in the LV-TPK RNAi-GFP infected cultured neurons was significantly lower at 7 days and 14 days after infection, as compared with neurons infected with LV-Ctrl-GFP (N = 4 & N = 3 culture preparations). **d** Lentiviral-mediated TPK RNAi significantly reduced TDP levels, but not that of thiamine monophosphate (TMP) and thiamine in cultured hippocampal neurons (N = 4 culture preparations). **e**, **f** Representative images of spines in cultured hippocampal neurons. Scale bar represents 5 µm. Quantitation of spine density in Ctrl, TPK RNAi and TPK RNAi + human TPK overexpression (hTPK Ovp) neurons (N = 3 culture preparations, n = 28, 26, 27 cells numbers, respectively). TPK knockdown significantly reduced the densities of dendritic spines in cultured hippocampal neurons (P < 0.05) and hTPK overexpression significantly rescued the effect of TPK knockdown on spine density (P < 0.01). All the data are expressed as mean ± SEM from three or four independent experiments. *P < 0.05, **P < 0.001, ***P < 0.0001, student’s t test and one-way ANOVA were used to determine the statistical significance of the differences

**Fig. 2:**
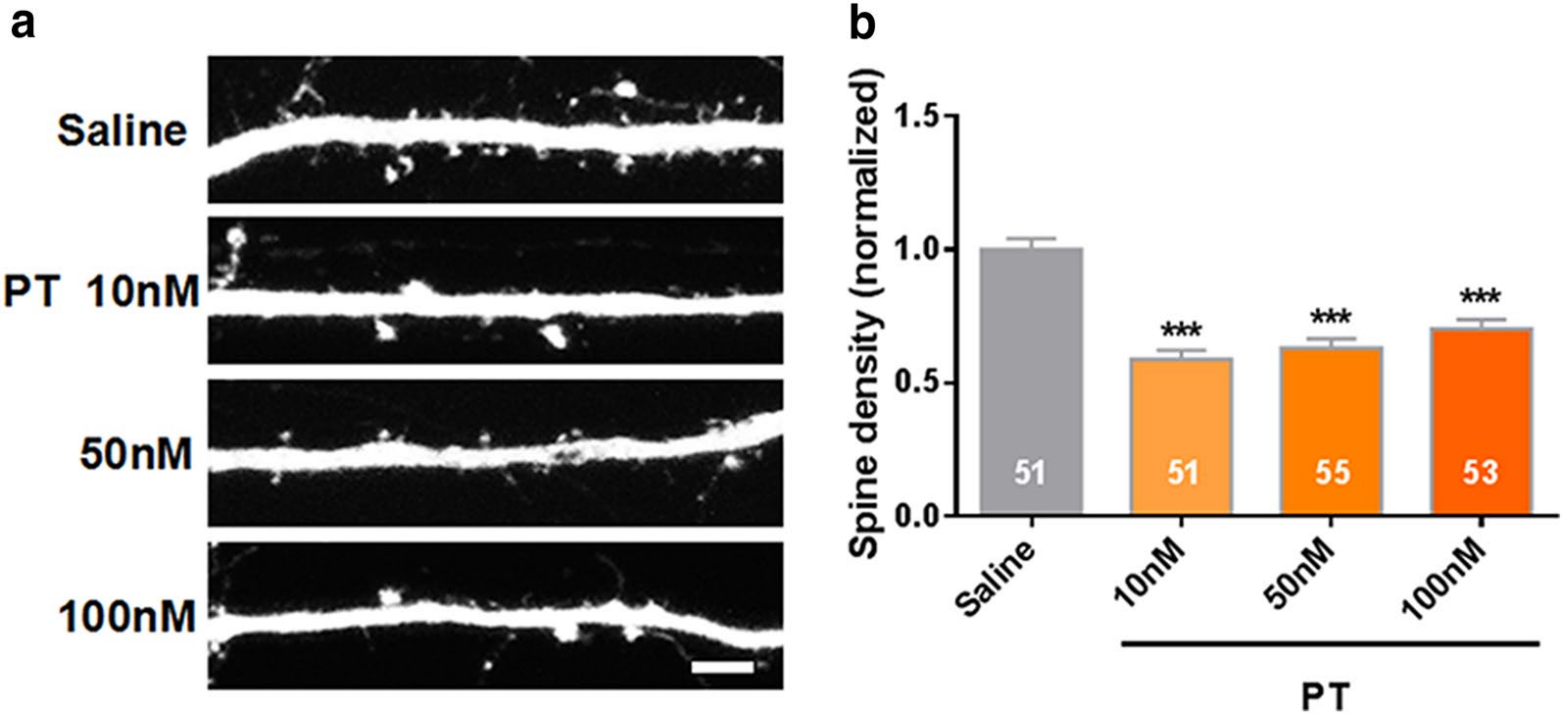
PT induced spine density decrease in vitro. **a** Increased concentration of pyrithiamine (PT) significantly decreased the dendritic spines density of culturing hippocampal neurons. **b** Quantification of spine density after the treatment with different concentrations of PT. (N = 3 culture preparations, n = 51, 51, 55, 53 cells numbers; P < 0.0001). All the data are expressed as mean ± SEM from three independent experiments. ***P < 0.0001, one-way ANOVA were used to determine the statistical significance of the differences

To reduce TDP levels in cultured hippocampal neurons, RNA interference (RNAi) of thiamine pyrophosphokinase (TPK), a key enzyme that phosphorylates thiamine into TDP, was used. As shown in Fig. 1a, mRNA level of TPK was significantly reduced in TPK RNAi oligo transfected N2A cell lines (P < 0.05, N = 3). The protein level of TPK was also reduced considerably after intervening with RNAi, (Fig. 1b). TPK mRNA level in the LV-TPK RNAi-GFP infected culturing hippocampal neurons was significantly lower at day 7 and day14 after infection, as compared with neurons infected with LV-Ctrl-GFP. (P < 0.05, N = 4 & N = 3, Fig. 1c). As shown in Fig. 1d, TPK RNAi significantly reduced TDP level in cultured hippocampal neurons (P < 0.05, N = 4), without affecting thiamine monophosphate (TMP) and thiamine levels (P > 0.05, N = 4). Importantly, the dendritic spines density of cultured hippocampal neurons was significantly lowered following TPK RNAi, an effect fully rescued by the overexpression of RNAi-resistant TPK (P < 0.05 and P < 0.001, N = 3, n = 28, 26, and 27, respectively, Fig. 1e, f). Consistently, pyrithiamine (PT), a TPK competitive substrate and TDP antagonist, significantly decreased the dendritic spines density of cultured hippocampal neurons (P < 0.05 and P < 0.001, N = 3, n = 51, 51, 55 and 53, respectively, Fig. 2a, b).

**Fig. 3:**
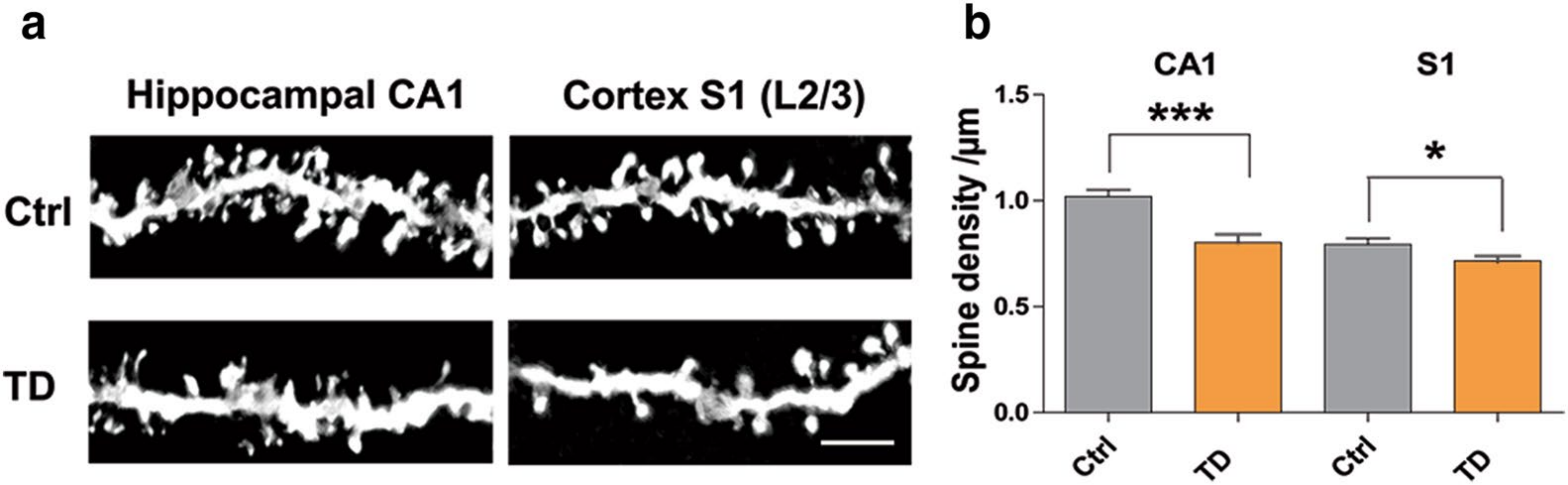
Thiamine deficiency induced spine density decrease in vivo. **a** Representative images of spines from hippocampal CA1 and cortex S1 regions of control mice and thiamine deficiency mice. Scale bars represent 5 µm. **b** Quantitation of spine density in CA1 and S1 L2/3 pyramidal neurons. Spine density in thiamine deficiency mice was significantly reduced as compared with that in control mice (N = 3 and 4 mice, n = 22, 26 cells numbers, P < 0.001 (CA1), and 30, 40 cells numbers, P < 0.05 (S1). All the data are expressed as mean ± SEM from three or four independent experiments. *P < 0.05, ***P < 0.0001, student’s t test and one-way ANOVA were used to determine the statistical significance of the differences

In vivo, mice with thiamine deficiency exhibited significantly reduced dendritic spine density in both hippocampal CA1 region (P < 0.001, N = 3 and 4, n = 22 and 26) and primary somatosensory cortex (P < 0.05, N = 3 and 4, n = 30 and 40, Fig. 3a, b), as measured by Golgi staining.

### Benfotiamine ameliorated Aβ induced spines density decrease

**Fig. 4:**
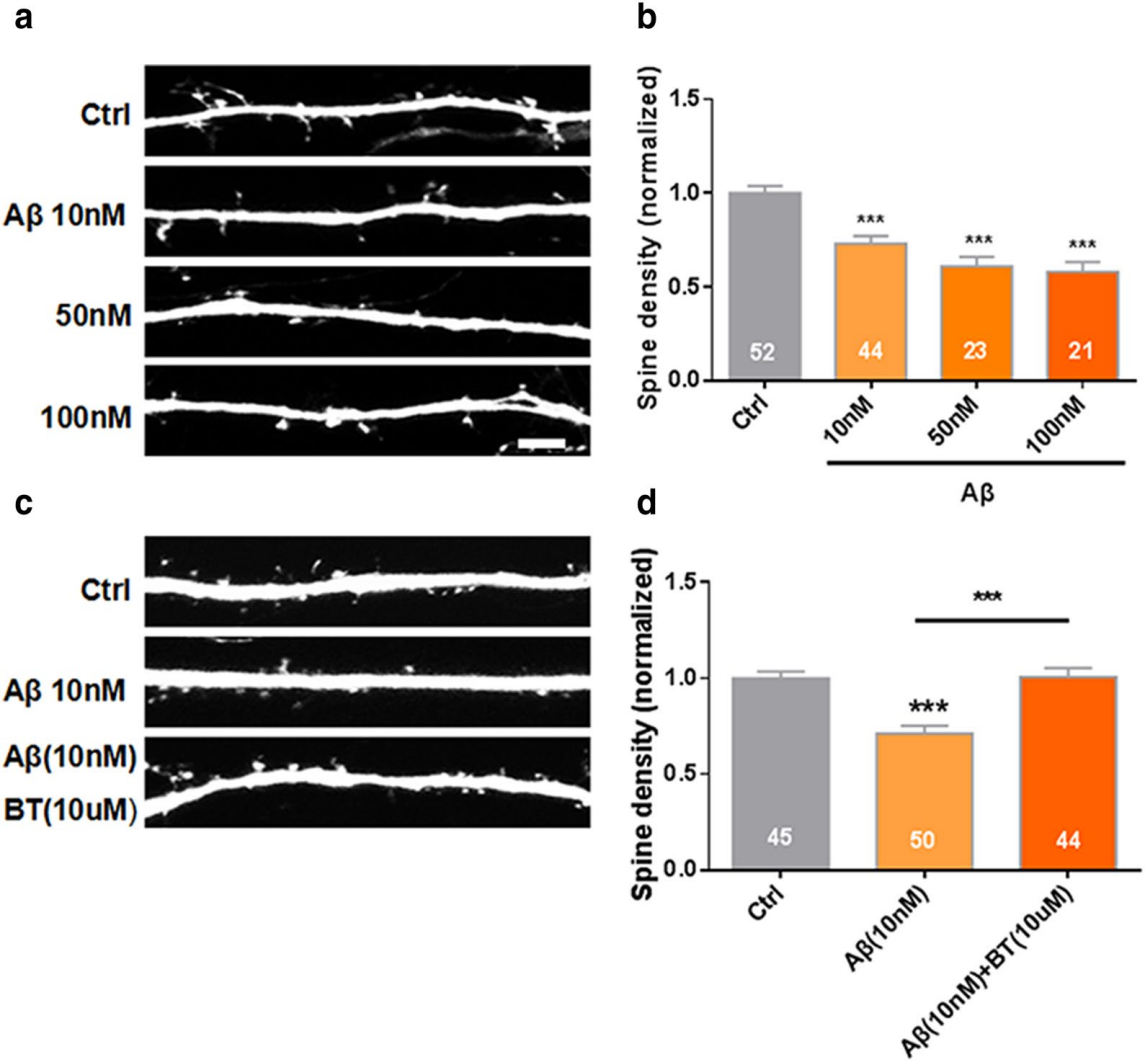
Benfotiamine ameliorated Aβ induced spines density decrease. **a** Oligomeric Aβ significantly decreased the dendritic spines density of cultured hippocampal neurons in a dose-dependent manner. **b** Quantification of spine density after Aβ incubation (P < 0.05 and P < 0.001, N = 3 culture preparations, n = 52, 44, 23 and 21 cells numbers). **c** Benfotiamine (BT) (10 μM) significantly ameliorated the reduction of Aβ (10 nM) induced spines density. **d** Quantification of spine density after Aβ and BT intervention (P < 0.05 and P < 0.001, N = 3 culture preparations, n = 45, 50, and 44 cells numbers). All the data are expressed as mean ± SEM from three independent experiments. *P < 0.05, **P < 0.001, ***P < 0.0001, one-way ANOVA were used to determine the statistical significance of the differences

Previous study showed that benfotiamine, a thiamine lipophilic derivative with better bioavailability, could alleviate the pathological alterations and cognitive dysfunction in APP/PS1 mice [27]. However, its effect on synapse loss was unknown. Oligomeric and fibrillar forms of amyloid-β (Aβ) peptide, one of the hallmarks of AD pathology, cause synaptic dysfunction and spine loss. Consistently, here we found that oligomeric Aβ in a dose-dependent manner significantly decrease dendritic spines density of cultured hippocampal neurons (P < 0.05 and P < 0.001, N = 3, n = 52, 44, 23 and 21, respectively, Fig. 4a, b). More importantly, benfotiamine significantly ameliorated Aβ induced spines density decrease (P < 0.05 and P < 0.001, N = 3, n = 45, 50, and 44, respectively, Fig. 4c, d).

### Thiamine deficiency led to lowered excitatory neurotransmission, and impaired hippocampal long-term potentiation

**Fig. 5:**
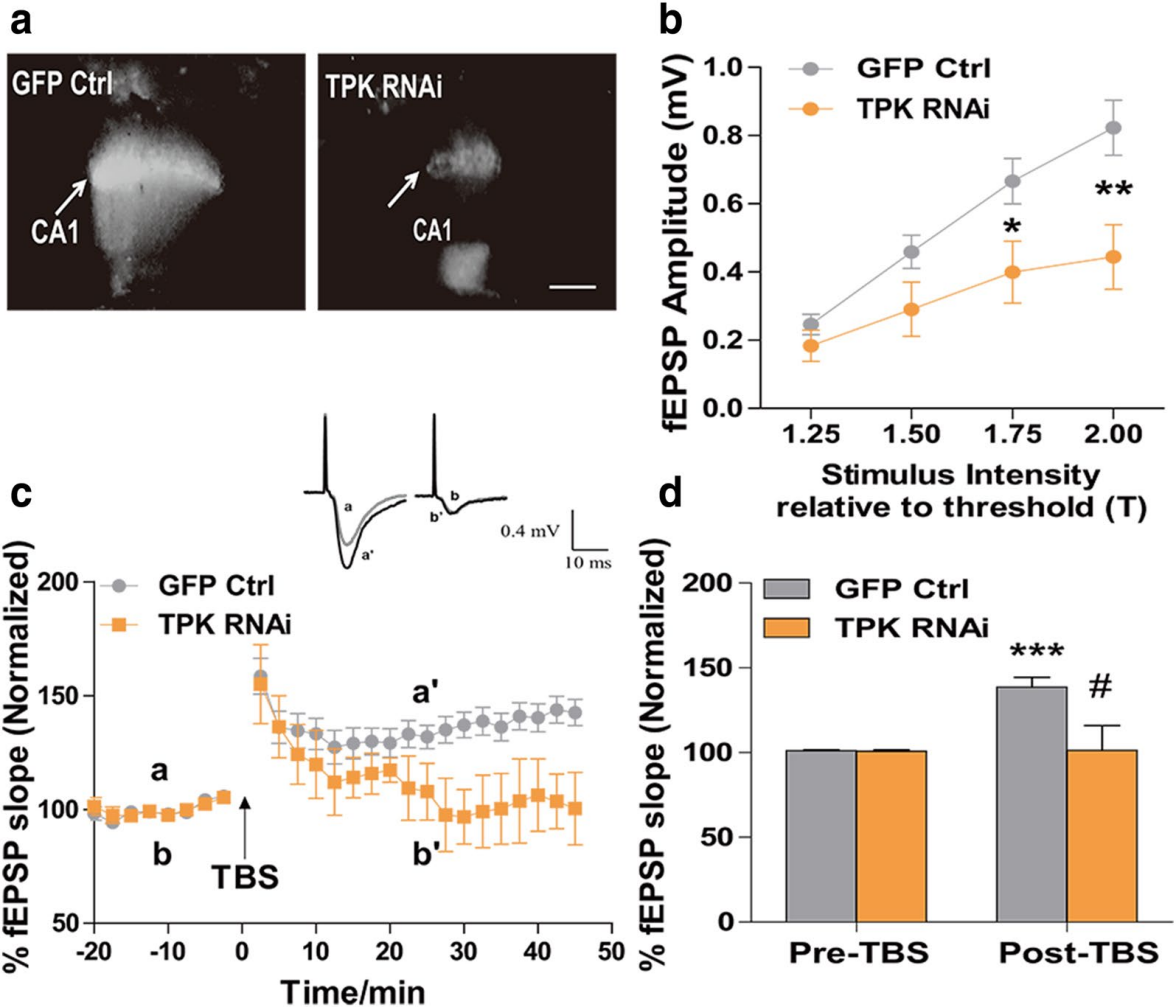
Thiamine deficiency led to lowered excitatory neurotransmission, and impaired hippocampal long-term potentiation. **a** Brain slices showed significant GFP fluorescence in the CA1 region 3 weeks after injection with either control lentivirus (GFP only) or that expressing TPK RNAi. Arrows indicate electrophysiological recording sites. Scale bar represents 200 µm. **b** Input–out curves of fEPSP amplitude for mice injected with control (n = 11 cells numbers) or TPK RNAi (n = 14 cells numbers; P < 0.05 at 1.75 and P < 0.05 at 2.00) lentivirus. **c** LTP was impaired in neurons infected with TPK RNAi (n = 5 cells numbers), as compared with GFP control (n = 10 cells numbers). The inserts showed the raw records of the fEPSP before (a, b) and after (a’, b’) LTP induction by TBS stimulation. **d** Histogram showing that LTP could not be induced in TPK RNAi neurons. **P < 0.001 vs baseline (pre-TBS); #P < 0.01 vs GFP control by two-way ANOVA and post hoc tests. All the data are expressed as mean ± SEM from three independent experiments. *P < 0.05, **P < 0.001, ***P < 0.0001, student’s t test and one-way ANOVA were used to determine the statistical significance of the differences

Measuring the input–output response of field excitatory postsynaptic potential (fEPSP) by evoking Schaffer collateral to CA1, we found that reducing brain TDP level through lentiviral-mediated TPK RNAi (Fig. 5a) significantly reduced the strength of hippocampal synaptic transmission (P < 0.05 and P < 0.01, n = 11 and 14, respectively, Fig. 5b). Synaptic plasticity is one of the most important properties of the mammalian brain as it underlies learning and memory processes. One of the main forms of synaptic plasticity found at the excitatory synapses is the long-term potentiation (LTP), which requires the activation of postsynaptic glutamate receptors (NMDA and AMPA) and is altered in neurodegenerative diseases [11]. We further measured synaptic plasticity in these neurons through theta burst stimulation (TBS)-induced LTP. Thirty minutes after TBS, LTP was successfully induced in control neurons infected with control vector, with the fEPSP slope increasing to 139 ± 5.75% (P < 0.001, n = 10). In contrast, LTP induction was significantly suppressed in 5 slices from TPK RNAi lentiviral infected neurons, indicating impaired synaptic plasticity after TPK knockdown (P < 0.05, Fig. 5c, d).

## Discussion

The current study explored the effects of TDP reduction on synaptic transmission and the function of neural cycle. We uncovered that TDP reduction significantly lessened the density of dendrite spines in vitro and in vivo, lowered excitatory neurotransmission, and impaired hippocampal LTP. It has long been verified that TDP reduction is widespread and serves as a promising biomarker for AD [7, 9]. Thus, the current results reveals that TDP reduction may be one of causal factors for the dysfunction of synaptic transmission in AD.

In terms of AD models in vitro. Apolipoprotein E ε4 (APOE 4), β-amyloid precursor protein (APP), presenilin 1 (PSEN1), or presenilin 2 (PSEN2) genes have been well reported in the pathogenesis of AD. Genome-editing applications such as CRISPR–Cas9 and induced pluripotent stem cell (iPSc) have been used in remodeling AD genetic mutations and disease progression [12, 13]. It is not surprising that synaptic dysfunction can be hardly found in such models, since neuronal maturation is one of the biggest challenges for researcher to overcome [14]. In this study, we established a new AD in vitro model induced by thiamine deficiency.

Glucose is a dominant substrate of brain energy metabolism, on which brain function absolutely depends. Generally, brain glucose hypometabolism in AD is considered as a consequence of synaptic and neuronal dysfunction or loss, as lower brain functionality requires less energy metabolism. However, the brain, as an organ with the most abundant energy consumption in the body, is vulnerable to the dyshomeostasis of glucose metabolism. Glucose metabolism predominantly takes place in mitochondria. Mitochondrial dysfunction has been well demonstrated as an early event in AD [15, 16]. Accumulating evidence suggests that mitochondrial perturbation is a key factor for synaptic failure and degeneration in AD. As a critical co-enzyme of mitochondrial enzymes pyruvate dehydrogenase and α-ketoglutarate dehydrogenase, once TDP is diminished in brain, mitochondrial function for glucose metabolism is disturbed [17] and brain energy metabolism is out of balance. Further, TDP reduction provokes multiple pathogenic factors, including oxidative stress [18], neuroinflammation [19], and enhanced activities of glycogen synthase kinase-3 [20] and β-secretase [21], which has been used to model AD-related neurodegenerative diseases. Thus, TDP reduction impairs the structure and function of synapse and neural cycle by perturbing brain energy metabolism and induces multiple pathophysiological cascades.

In our previous study, by Y-maze test in TD mouse model, we found cognitive dysfunction, which was induced by TD [22]. In addition, spatial cognitive impairment was also found in pyrithiamine-induced thiamine deficiency (PTD) male rats via water-maze testing [23]. Vedder and his colleagues also validated significant cognitive impairment in TD rats [24]. Therefore, TD has been well demonstrated with cognitive dysfunction in most of the relevant studies.

Aβ plays a major role in the pathogenesis of AD. It is demonstrated that Aβ_1–42_ oligomers exhibits neurotoxicity that can cause synaptic dysfunction [25]. In this study, we repeated the previous results that Aβ damages the density of dendritic spines in a dose-dependent manner. Importantly, benfotiamine, a thiamine lipophilic derivative with better bioavailability, significantly rescued the reduced density of dendritic spines caused by Aβ. Such results strongly parallel to the study which has been done by Pan et al. that benfotiamine considerably ameliorated cognitively impairment in APP/PS1 mice as well as in mild to moderate AD patients [26, 27]. These results suggest that the modulation of brain glucose metabolism and its associated pathophysiological cascades is a potential target for AD therapy.

## Conclusions

TDP reduction can lead to the abatement of dendrite spine density in vitro and in vivo, lowered excitatory neurotransmission, and impaired hippocampal LTP. It may be one of causes of synapse and neural circuit defects in AD. Our study promotes the understanding of AD brain dysfunction and provides new insight into disease-modifying therapy.

## Methods

### Animals

All animal care and experimental procedures were approved by Medical Experimental Animal Administrative Committee of Fudan University and by the Institutional Animals Care and Use Committee of the Institute of Neuroscience, Shanghai Institutes for Biological Science, Chinese Academy of Science. Two-month-old C57BL/6 male mice were used for thiamine deficiency experiments. Sprague–Dawley rats of postnatal day 0 (P0) were used for culturing hippocampal neurons preparation. All animals were housed in a humidity- and temperature-controlled environment with 12-h light/dark cycles and free access to food and water. Thiamine deficiency in mice was induced by feeding a thiamine-depleted diet (Bio-serv Company, USA) and distilled water ad libitum for up to 26 consecutive days. Control animals received a thiamine-containing diet (Bio-serv Company, USA) and distilled water ad libitum. Precisely, for the thiamine deficiency model in vivo, we used 3 mice as TD group and 4 mice as control group. For the fEPSP amplitude test, we used 11 mice for the injection of control lentivirus and 14 mice for the injection of RNAi lentivirus.

### Protein purification and western blots

Brain tissues, cultured neurons, or cell lines were lysed with radioimmunoprecipitation assay (RIPA) lysis buffer, containing protease inhibitor mixture and PhosSTOP phosphatase inhibitor cocktail (Roche). Protein concentrations were determined with the Pierce™ BCA protein assay kit according to the manufacturer’s instruction (Thermo Scientific, Rockford, IL). The same amount of protein was loaded in each lane for electrophoresis on a 10% denaturing Tris/glycine sodium dodecyl sulfate (SDS) polyacrylamide gel. Polyvinylidene fluoride membranes (Millipore) were blocked in 5% milk in Tris-buffered saline supplemented with 0.1% Tween-20 (TBS-T, pH 7.4) for 2 h. Proteins were transferred to the membrane and incubated with primary antibody overnight: TPK1 (Proteintech, 10942-1-AP, 1:1000), HA (Abcam, AB9110, 1:1000), GAPDH (KangChen, KC5G4, 1:10,000), Tubulin (Santa Cruz, sc-101815, 1:500) in 3% milk TBS-T. Membranes were washed in TBS-T and incubated for 2 h with horseradish peroxidase (HRP)-conjugated anti-mouse or anti-rabbit IgG (Millipore) in 3% milk TBS-T. Results were quantified using Image J (N.I.H.).

### Culture and transfection of hippocampal neurons and N2A cells

Hippocampal neurons were cultured from postnatal day 0 (P0) Sprague–Dawley rats, essentially as previously described [28]. Briefly, neurons were plated on PDL (P6407, Sigma-Aldrich, St. Louis, MO, USA)-coated glass coverslips (63-3009, Assistent, Sondheim/Rhön, Germany), at approximately 50,000 cells/cm^2^, in Neurobasal medium (10888-022, Thermo Fisher Scientific, Waltham, MA, USA) supplemented with B-27 (17504-044, Thermo Fisher Scientific), 2 mM Glutamax-I (35050-061, Thermo Fisher Scientific), and 2.5% FBS (SH30070.03, GE Healthcare Life Sciences, Pittsburgh, PA, USA). On the third day in vitro (DIV 3), cells were treated with the mitotic inhibitor 5-fluoro-2′-deoxyuridine (F0503, Sigma). Calcium phosphate transfection was carried out at DIV 5–7 using 2–4 μg of DNA/well. GFP was included as a morphology marker. Spine density was normalized to control GFP-transfected culture from the same preparation. At least three independent culture preparations were assayed. N2A cells were cultured in high glucose Dulbecco’s modified Eagle medium (DMEM) supplemented with 10% FBS (SH30070.03, GE Healthcare Life Sciences) and transfected using Lipofectamine 2000 (11668019, Thermo Fisher Scientific). For pharmacological treatment, neurons were treated with 50 μM pyrithiamine (PT, Sigma-Aldrich), 10, 50, and 100 nM Aβ (SCP0030, Sigma-Aldrich) and 10 μM benfotiamine (BT, Rixin biological technology co., LTD, Shanghai) on DIV 12 and fixed on DIV 14. Pyrithiamine, Aβ, and benfotiamine were dissolved in saline, and control neurons were treated with an equal volume of saline.

### Constructs

TPK RNA interference (RNAi) was generated in pSuper-GFP using the following sequence GAAGGGCTGTGATCTTATTTT, which targets both mouse (NM_013861) and rat (NM_001134994) TPK. The same sequence was used to generate TPK RNAi lentivirus (packaged by GeneChem Technology, Shanghai, China). Mouse TPK (NM_013861) and human TPK (NM_022445) constructs were generated by cloning their full coding sequences into pCS2-myc.

### Real-time qPCR

Total RNA was extracted from culture neuron using TRIzol reagent (Invitrogen). For Real-time PCR experiments, complimentary cDNA was synthesized using the M-MLV reverse transcriptase (Promega) according to the manufacturer’s protocol. The following primers were used: 5′-CTGCCCAGAACATCATCCCT-3′ (forward) and 5′-TGAAGTCGCAGGAGACAACC)-3′ (reverse) for *Gapdh*; 5′-ACACCAAGAAGGGCTGTGAT-3′ (forward) and 5′-TCTGTGCTTTCCTGGTTGGAG-3′ (reverse) for *TPK*. Real-time PCR was performed using SYBR Green Master Mix (TaKaRa, Japan) on LightCycler 480 (Roche Applied Science). All reactions were performed in triplicates, and the results were normalized to that of control groups from the same culture preparation.

### Golgi-Cox staining and imaging analysis

Mice were deeply anesthetized by intraperitoneal injection of 0.7% sodium pentobarbital solution. The brain was immediately removed, rinsed in PBS, and stained using Golgi-Cox method, according to the manufacturer’s instructions (Rapid Golgi; FD NeuroTechnologies). Briefly, brain tissue was stored at room temperature for 8 days in the dark, before coronal Sects. (150 µm) were cut using a Leica CM1900 cryostat (Wetzlar, Germany). Images (Z stacks at 1 µm intervals) were acquired on a Zeiss Pascal laser scanning microscope (Jena, Germany) with a 63× oil-immersion Neofluor objective (N.A. = 1.4). Secondary basal dendritic segments from hippocampal CA1 and cortical S1 regions were imaged. “n” presents the number of neurons and at least three mice were analyzed per experimental condition. Dendrite length was measured using ImageProPlus software (MediaCybernetics). Spine densities were calculated as the mean number of spines per micrometer dendrite. All images were analyzed blinded to the experimental condition and as it were acquired. For Golgi staining example images, bright-field Z-stack images were projected at minimal intensity and inverted, background was then subtracted, followed by brightness/contrast adjustment within linear ranges, using ImageJ (NIH).

### Electrophysiological study

Mice were randomly assigned to TPK RNAi-GFP or GFP control groups. After mice were anesthetized with sodium pentobarbital (0.1 mg/g, intraperitoneal) and placed in a stereotaxic frame, a microsyringe was used to inject solution containing TPK RNAi-GFP (5 × 10^8^ TU/ml) or GFP lentivirus (5 × 10^8^ TU/ml) into the left CA1 area in the dorsal hippocampus. All mice were allowed for recover for 3–4 weeks before electrophysiological recordings. For brain slice preparation, mice were anesthetized with sodium pentobarbital and intracardially perfused with cold cutting solution through the left ventricle. After mice were then decapitated, brains were rapidly dissected out and placed in ice-cold cutting solution (215 mM sucrose, 26 mM NaHCO_3_, 20 mM glucose, 2.5 mM KCl, 1.6 mM NaH_2_PO_4_, 4 mM MgSO_4_, 4 mM MgCl_2_, and 1 mM CaCl_2_). Hippocampal slices were cut at 300 µm with a vibratome (VT1200S, Leica, Germany) and transferred to a transitional chamber containing artificial cerebrospinal fluid (ACSF, composed of 119 mM NaCl, 2.3 mM KCl, 1.3 mM MgSO_4_, 26.2 mM NaHCO_3_, 1 mM NaH_2_PO_4_, 2.5 mM CaCl_2_, and 11 mM glucose; bubbled with 95% O_2_/5% CO_2_, pH ~ 7.4).

Hippocampal slices containing GFP signal in the CA1 region were used in this study. The strength of synaptic transmission was determined by measuring the maximum slope of the fEPSPs in the stratum radiatum of CA1 region evoked by stimulating the Schaffer collateral with a bipolar tungsten electrode and glass microelectrodes filled with ACSF with 3–7 MΩ resistance. To determine the input–output relationship, a series of ascending stimuli were delivered in each hippocampus slice. To standardize the stimulus intensity, the threshold of stimulus intensity (T) was first determined, then the corresponding fEPSP amplitudes of 1.25 × T, 1.5 × T, 1.75 × T, and 2 × T were obtained, and the input–output relation (I–O) was plotted. For LTP experiments, only slices with maximal evoked fEPSPs amplitude over 0.5 mV were used. fEPSPs were evoked by pulses at a 0.033 Hz stimulation frequency and with an intensity that was elicited a 40–50% the maximal fEPSP response. After at least 30 min of stable baseline recording, LTP was induced by a Theta burst stimulation (TBS, 4 pulses at 100 Hz, 15 trains in 0.2 s interval) using the same stimulus intensity as for baseline recording. After LTP induction, fEPSPs were recorded for 45 min.

### Statistical analysis

SPSS software (version 18.0; SPSS Inc) and Graphpad Prism 6 (version 6.01; GraphPad software) were used for statistical analyses. Student’s t-test for single comparisons or one-way ANOVA for multiple comparisons with appropriate Tukey’s or Dunnett’s Multiple Comparison tests were used to identify statistical differences. Results shown represent the mean ± SEM. “n” refers to the number of cells and “N” refers to the number of animals or experiments, unless otherwise indicated. All conditions statistically different from its control are indicated, where * represents P < 0.05, **P < 0.01, and ***P < 0.001.

## Abbreviations

Aβ: amyloid-β
AD: Alzheimer’s disease
ApoE: apolipoprotein E
BT: benfotiamine
fEPSP: field excitatory postsynaptic potential
GSK-3β: glycogensynthetase kinase-3β
hTPK: human TPK
LTP: long-term potentiation
PT: pyrithiamine
RNAi: RNA interference
TBS: theta burst stimulation
TDP: thiamine diphosphate
TPK: pyrophosphokinase
